# Counter-stereotypical pictures as a strategy for overcoming spontaneous gender stereotypes

**DOI:** 10.3389/fpsyg.2015.01291

**Published:** 2015-08-27

**Authors:** Eimear Finnegan, Jane Oakhill, Alan Garnham

**Affiliations:** ^1^School of Psychology, University of SussexBrighton, England; ^2^School of Psychological Sciences and Health, University of StrathclydeGlasgow, Scotland

**Keywords:** pictures, spontaneous, gender, stereotype, reduction

## Abstract

The present research investigated the use of counter-stereotypical pictures as a strategy for overcoming spontaneous gender stereotypes when certain social role nouns and professional terms are read. Across two experiments, participants completed a judgment task in which they were presented with word pairs comprised of a role noun with a stereotypical gender bias (e.g., beautician) and a kinship term with definitional gender (e.g., brother). Their task was to quickly decide whether or not both terms could refer to one person. In each experiment they completed two blocks of such judgment trials separated by a training session in which they were presented with pictures of people working in gender counter-stereotypical (Experiment 1) or gender stereotypical roles (Experiment 2). To ensure participants were focused on the pictures, they were also required to answer four questions on each one relating to the character’s leisure activities, earnings, job satisfaction, and personal life. Accuracy of judgments to stereotype incongruent pairings was found to improve significantly across blocks when participants were exposed to counter-stereotype images (9.87%) as opposed to stereotypical images (0.12%), while response times decreased significantly across blocks in both studies. It is concluded that exposure to counter-stereotypical pictures is a valuable strategy for overcoming spontaneous gender stereotype biases in the short term.

## Introduction

While English has a number of personal nouns that include maleness or femaleness as part of their lexical definitions (e.g., father, girl, son), or are formally marked for lexical gender through the use of suffixes (e.g., waitress, landlord, landlady), the majority of human nouns in English are not gender specific. Instead, gender information associated with a human noun is typically indicated through social gender. This term refers to stereotypical assumptions about appropriate male and female social roles and the extent to which those roles are filled by females or males (Hellinger and Bußmann, 2001). Indeed social gender is now more commonly referred to as gender (stereo)typicality and is simply defined as the likelihood of a noun referring to women or men (Irmen and Roßberg, 2004). This gender typicality plays an important role in building cognitive representations of gender and is the reason why people come to expect, for example, surgeons to be male and nurses to be female. It is now well established that such occupational stereotypes are activated spontaneously and unintentionally when certain gender-biased role nouns are read (Carreiras et al., 1996; Garnham et al., 2002, 2012; Kennison and Trofe, 2003; Duffy and Keir, 2004; Reynolds et al., 2006; Irmen, 2007; Kreiner et al., 2008; Finnegan et al., 2015), thus contributing to the maintenance and propagation of gender stereotypes in English speakers.

Once stereotypes or prejudiced associations are established, they can start to function automatically (Bodenhausen et al., 2009). Automatic processes typically require few attentional resources and are activated spontaneously, often without the perceiver’s control or awareness (Bargh, 1994). Moreover, these associations can be activated independently of a person’s conscious endorsement of them (Bodenhausen et al., 2009). In contrast, controlled processes operate through conscious intent and involve the attention of the perceiver (Schneider and Shiffrin, 1977). Both types of process are relevant to the current research in which we investigate a strategy for overcoming the spontaneous activation of occupational gender biases so as to ultimately result in lower levels of stereotype application.

Given the subtle pervasiveness of stereotype biases and prejudice, researchers have focused attention on devising means to overcome them. Indeed, Shiffrin and Schneider (1977) argued that with considerable and consistent training automatic responding to a particular stimulus could be “unlearned” and newer responses trained to take their place (Schneider and Shiffrin, 1977). While biased associations have proven difficult to completely overturn, they have proven malleable given appropriate strategies and conditions (see Blair, 2002 for a review). Evidence has been mounting for the positive effect of counter-stereotype promotion in tackling stereotype biases, because such stereotype incongruent information appears to weaken the stereotype itself or access to it (e.g., Kawakami et al., 2000; Blair et al., 2001; Dasgupta and Greenwald, 2001; Lai et al., 2014). Thus, in this article we explore how strengthening counter-stereotype associations may reduce gender stereotyping in relation to occupational role nouns.

While stereotype representations reflect the strongest or most typical group associations, research suggests that these representations may also include information about counter-stereotypes. For instance, it has been shown that people represent subtypes that are inconsistent with a group stereotype, for example that of business woman or female athlete (Devine and Baker, 1991; Green and Ashmore, 1998; Coats and Smith, 1999). Blair et al. (2001) argued that as stereotypes and counter-stereotypes are often polar opposites, it is unlikely that they would be represented completely independently of one another. Indeed increasing the accessibility of one of these constructs could result in a decrease in accessibility of the other (e.g., Dijksterhuis and van Knippenberg, 1996).

Blair et al. (2001) explored whether increasing the accessibility of counter-stereotypes through use of a mental imagery task could result in lower levels of implicit gender stereotype activation. They devised an experiment with four different imagery conditions: stereotypic (participants imagined a weak woman), counter-stereotypic (participants imagined a strong woman), gender neutral (participants imagined a holiday in the Caribbean) and no imagery (participants played with a simple water game for 5 min; Experiment 2; Blair et al., 2001). Implicit stereotypes were measured both before and after the 5 min mental imagery task using the Implicit Association Test (IAT; Greenwald et al., 1998). Participants in the counter-stereotype condition subsequently produced significantly weaker implicit gender stereotypes than those in the three other mental imagery conditions, thus providing convincing evidence for the moderating effect of counter-stereotype mental imagery on implicit stereotypes. Indeed the same pattern of results was found when this mental imagery strategy was used with two further measures of stereotype bias; the Go/No-go association test (GNAT; Experiment 4) and a false memory paradigm (Experiment 5).

This comprehensive set of experiments by Blair et al. (2001) suggests that implicit associations can be altered by directing participants’ attention to subtypes of group members or triggering counter-stereotypical links in the cognitive network (Kunda and Thagard, 1996; Bodenhausen and Macrae, 1998). A more direct approach to increasing counter-stereotype saliency was taken by Kawakami et al. (2000) who devised a non-stereotypic association training method aimed at reducing automatic stereotyping toward racial groups and skin heads. This association training involved presenting participants with counter-stereotypic and stereotypic word pairs relating to the category of interest. The task was to repeatedly affirm (i.e., say ‘yes’) and negate (i.e., say ‘no’) the counter-stereotypic and stereotypic pairings respectively. It was found that participants who received extensive training in counter-stereotype affirmation/stereotype negation showed lower levels of automatic stereotyping on a primed Stroop task (Studies 1 and 2) and a person categorization task (Study 3) than those who received little or no training.

Given the extent of research aimed at overcoming stereotypes and prejudice, it is important to compare the efficacy of interventions that have led to significantly lower levels of bias. Estimates of effect magnitude are critical to such comparisons, yet are rarely included in stereotype or prejudice reduction research, where null hypothesis significance testing is dominant. Instead, researchers tend to provide evidence that an intervention results in less implicit prejudice or stereotyping than a control condition (Frick, 1996; Lai et al., 2013). Lai et al. (2014) sought to address this issue by holding a research contest to experimentally compare 17 interventions aimed at overcoming implicit racial preferences. These interventions fell into six categories of which “exposure to counter-stereotypical exemplars” proved the most effective (*d* = 0.38, 95% CI [0.32, 0.44]). The three most successful strategies within this category involved getting participants to imagine a vivid counter-stereotypic scenario, shifting group boundaries through competition (i.e., cooperating with outgroup members and competing against ingroup members in a dodgeball game), and getting participants to practice an IAT with counter-stereotypical exemplars (i.e., positive Black exemplars, and negative White exemplars)^1^.

While Lai et al.’s (2014) research was aimed at overcoming implicit racial biases (as opposed to the spontaneous gender biases that we are interested in), their findings are relevant here as stereotype reduction strategies have often proved successful across different domains. For instance, social norm information has been used to successfully reduce biases against groups such as racial minorities (e.g., Stangor et al., 2001), people suffering from obesity (Puhl et al., 2005) and has also lowered levels of spontaneous gender stereotyping (Finnegan et al., 2015).

Broader support for the use of counter-stereotypes in bias reduction is found with Bodenhausen et al. (2009), who advocate the role of diverse environments in undermining biased representations. Evidence suggests that increased interaction with out-group members can substantially weaken biased attitudes, and automatic negative emotional and physiological reactions to these outgroup members (Blascovich et al., 2001; Pettigrew and Tropp, 2006). Similarly, diverse environments can influence automatic stereotypes and attitudes about groups, as it has repeatedly been found that counter-stereotype exemplars of devalued groups can result in more positive attitude and stereotype activation in both lab (e.g., Dasgupta and Greenwald, 2001; Joy-Gaba and Nosek, 2010) and real-world settings (e.g., Dasgupta and Asgari, 2004). For instance, by exposing participants to admired African American individuals and disliked European Americans, implicit preference for Whites compared to Blacks was successfully reduced on an IAT (Dasgupta and Greenwald, 2001).

But how do counter-stereotypes operate to reduce levels of stereotyping? Two potential processes are (1) the bookkeeping process in which stereotypes are hypothesized to change slowly, through encountering numerous counter-stereotype exemplars of a particular category and (2) the conversion process in which stereotypes are thought to change more rapidly, upon encountering fewer, yet more striking counter-stereotype exemplars than postulated in the book-keeping process (Operario and Fiske, 2004). While both of these processes highlight means of achieving stereotype reduction via counter-stereotype information, a third process, subtyping, suggests how such information could work to protect the stereotype. Essentially, subtyping processes may ensure that the original stereotype remains unchanged as new categories are formed to account for counter-stereotype information. However, it is also possible that stereotypes could be weakened and reduced with sufficient category variation and subtyping (Operario and Fiske, 2004).

In the current research, Experiment 1 employs a striking counter-stereotype strategy in which participants are presented with pictures of men and women working in obviously counter-stereotypic roles. We hypothesize that these gender-salient pictures will bring about stereotype reductions through direct and immediate conversion processes. By highlighting category variability we hope to strengthen counter-stereotype representations and remind participants that, for example, a surgeon could be female and a nurse male. This salient counter-stereotypical information should be incorporated into the perceiver’s gender and occupation-related cognitive representations so as to update and modify them. The measure of stereotyping that we used in conjunction with this picture training was a judgment task devised by Oakhill et al. (2005).

Oakhill et al. (2005) were interested in whether gender biases are evoked for single words, and the extent to which these biases can be overcome. To explore this question they asked participants to quickly decide whether two terms presented onscreen could refer to one person. These word pairs comprised a role noun that was stereotype biased (e.g., *builder, beautician*) and a kinship term that was definitionally gendered only (e.g., *uncle, aunt*). In order to respond successfully, participants needed to take definitional gender into account (e.g., that an uncle is always male) but to dismiss stereotypical gender (e.g., that most beauticians are female).

Across a series of studies, Oakhill et al. (2005) found that participants consistently rejected stereotype incongruent pairings (e.g., *builder/mother*) to a significantly greater extent than stereotype congruent pairings (e.g., *builder/father*). This was still the case when they were explicitly reminded that nowadays many jobs are not marked for gender and that they should carefully consider whether the first term presented (i.e., the role noun) could be occupied by men, women or both (Experiment 4). Results of this research provide strong evidence that there is an automatic component to responding, as participants struggled to overcome the gender stereotype information associated with the role nouns, even when its activation was detrimental to task performance. Indeed the authors posit that such gender stereotype information is incorporated immediately, and likely automatically, into a perceiver’s mental representation.

This research has much in common with that of Finnegan et al. (2015) who sought to overcome such occupational gender biases through the use of social-consensus feedback (again in conjunction with the judgment task of Oakhill et al., 2005). Finnegan et al. (2015) administered three blocks of judgment trials with social consensus feedback provided after each response in Block 2 only. This feedback consisted of a sentence stating the percentage of previous students at the university who had completed the judgment task and agreed with the participant’s choice, e.g., ‘*_% of previous students agreed with you.’* In reality this feedback was fictitious and constructed so as to strongly and consistently suggest that past participants did not succumb to stereotype biases, i.e., that they accepted stereotype incongruent pairings without a problem. In this way, the social feedback sought to reinforce non-stereotypic responding and highlight any discrepancy between a participant’s response and the peer group norm.

Performance on judgments of stereotype incongruent pairings was found to improve significantly following the introduction of social feedback in Block 2. Moreover, this improvement continued in Block 3 when the feedback was no longer given (Experiment 1), thus providing evidence for the use of social consensus information as a useful stereotype reduction strategy.

Other strategies aimed at overcoming gender biases for occupational role nouns have more frequently been examined in sentence comprehension studies. In such cases, a stereotyped term is typically followed by gender congruent or incongruent information in a match/mismatch paradigm, e.g., the *surgeon* went to work early as *he/she* was very busy. Processing difficulty is frequently evident in the incongruent condition relative to the congruent condition as the reader struggles to reconcile the unexpected definitional gender information on the pronoun with the stereotype-biased gender already generated by the occupational role term (e.g., Carreiras et al., 1996; Garnham et al., 2002; Kennison and Trofe, 2003; Duffy and Keir, 2004; Irmen, 2007; Kreiner et al., 2008; Garnham et al., 2012). However, such gender biases have successfully been overcome through establishing the sex of a character *before* a role noun is encountered, e.g., after reminding *himself/herself* about the letter, the *minister* immediately went to the meeting at the office (e.g., Duffy and Keir, 2004; Kreiner et al., 2008; Lassonde and O’Brien, 2013).

We report two studies in which we investigated the influence of counter-stereotype pictures as means of increasing counter-stereotype saliency and reducing levels of gender-based occupational stereotyping on the judgment task of Oakhill et al. (2005).

### Overview of Studies

In Experiment 1 participants were presented with two blocks of stereotype judgment trials, with the picture task immediately following the first block. In the picture task participants were presented with 24 pictures of people working in counter-stereotypical roles. The participants’ task was to answer a set of four questions for each picture about the character’s supposed earnings, leisure activities, job satisfaction and personal life. This was intended to result in deeper processing of the character presented and the counter-stereotypical job this person was depicted as holding.

It was hypothesized that participants would initially respond more slowly and less accurately to trials of stereotype incongruent word pairs (e.g., *nurse/father*) than to stereotype congruent word pairs (e.g., *nurse/mother*) in Block 1. However, following the picture training, it was hypothesized that the processing cost associated with the stereotype incongruent condition in Block 1 would be attenuated and lead to higher accuracy and faster reaction times to the critical trials in Block 2.

Experiment 2 was a control study which differed from Experiment 1 solely in the picture task. Participants were now presented with images of people working in stereotypical roles (as opposed to counter-stereotypical) to provide a clear basis for explaining the Block 1 to Block 2 changes in performance in Experiment 1. For these studies, 24 pairs of pictures of men and women working in the same occupational roles were first required, for instance, a female make-up artist (stereotypical) and a male make-up artist (counter-stereotypical). These pictures were collected through a web search and from a picture database. A short pilot study was conducted to evaluate (a) the similarity of the male and female versions of the pictures and (b) how realistic the pictures looked.

## Experiment 1

### Pilot Study

Twenty students (10 male and 10 female) took part in the pilot study that lasted 5 min. Each of the 24 picture pairs was presented as pictures of men and women working in the same roles. The participants’ first task was to rate these pairs on “how similar they are (ignoring gender and thinking about features such as the race, age, facial expression of the people, pose and the background).” Ratings were made on a scale ranging from 1 (very similar) to 6 (very dissimilar).

Next, the pictures were re-presented to the participants, who judged how realistic they found the pictures to be – again ignoring gender and thinking about features such as the race, age, facial expression of the people, pose and the background. In this part of the pilot study the 48 pictures were rated individually on a realism measure from 1 (very realistic) to 6 (very unrealistic).

The mean similarity rating across picture pairs was 2.24 (SD = 1.26), thus falling between the points of moderately similar (2) and mildly similar (3). The mean rating of how realistic a picture looked was 1.93 (SD = 1.26), thus falling between the points of very realistic (1) and moderately realistic (2). In two instances males and females were found to have significant differences in their ratings of similarity and in one instance had significant differences in their ratings of realism. Ultimately, however, all pictures were kept for the experimental task, as none were rated as being more dissimilar than similar or more unrealistic than realistic. Furthermore, because no obviously dissimilar or unrealistic pictures were included for rating, participants may have been stricter in their judgments than otherwise expected.

### Method

#### Participants

The participants were 30 monolingual native English speakers (14 male, 16 female) from the student population of the University of Sussex. Participants’ ages ranged from 18 to 37 years (*M*: 20.27; SD: 4.12) and they received either £6 or 4 course credits for taking part in the session, which lasted ∼45 min. Ethical approval for both experiments in this paper was obtained from the University of Sussex, School of Psychology Research Ethics Committee, which follows the British Psychological Society guidelines for ethics on human subject testing. All participants signed a consent form prior to participating.

### Materials

#### Gender-Biased Role Nouns

Gender biased role nouns were selected from norms compiled by Gabriel et al. (2008). The chosen items were the 12 most highly male-biased nouns (e.g., bricklayer), the 12 most highly female-biased (e.g., beautician), and the 12 closest to the neutral point on the scale (e.g., pedestrian). As described in Finnegan et al. (2015), the range of the bias ratings for the male terms is narrower than for the female items (11.10% vs. 17.55% respectively), while ratings of the neutral terms have the shortest range of 5.29%. These figures suggest that the neutral terms should prove less problematic for participants than the other role nouns. See Finnegan et al. (2015) for a full list of the stereotyped items, their associated bias ratings and all filler items.

#### Kinship Terms

As in previous studies, six kinship terms (three male, three female) were selected to be used as one of the terms in the word pairs (Oakhill et al., 2005; Finnegan et al., 2015). These terms were *father, mother, brother, sister, uncle, aunt*. Importantly, these words incorporate a specific gender into their definitions, e.g., the term ‘*brother*’ can only refer to a person of male sex.

#### Critical Word Pairs

The 12 male-biased, female-biased, and neutral role nouns were each combined once with the six kinship terms to produce a set of stereotype congruent (e.g., *pilot/brother, nurse/sister*), stereotype incongruent (e.g., *pilot/sister, nurse/brother*) and neutral word pairs (e.g., *artist/brother, artist/sister*). There were, therefore, 72 word pairs in each of the three congruency conditions, totaling 216 trials.

#### Filler Trials

Filler items were 240 word pairs created by pairing the six kinship terms with role nouns that are also gender-specific by definition (e.g., geisha, hero). In this way, filler trials were gender unambiguous pairings to which participants could respond with relative ease and certainty. These items were selected from norming studies conducted by Kennison and Trofe (2003) and Hamilton (2008).

#### Item Overview

The word pairs used in this study were identical to those of Finnegan et al. (2015) in content although the number of pairings presented differed. While that study had three blocks of trials (and a total of 456 word pairs) the current work used two blocks of trials (and a total of 304 word pairs). Therefore, use of the three original blocks from Finnegan et al. (2015) was counter-balanced so that each of their 456 pairs appeared an equal number of times across participants in the current experiments. This procedure also ensured that each of the six kinship terms appeared with each of the role nouns an equal number of times. In total, 184 items were intended to elicit a *yes* response (including all critical items) while 120 required a *no* response.

#### Picture Task

Twenty four pictures of a man or a woman working in a counter-stereotypical job environment were selected. Half of the pictures depicted people working in roles that were also mentioned in the judgment task and half depicted ‘new’ role terms that the participants had not yet been exposed to (six male and six female stereotypical terms in each case)^2^.

When displayed on-screen, the pictures were accompanied by two short sentences. These sentences always introduced the character in the picture and their job, e.g., “This is Rebecca. She is a bricklayer” or “This is Christopher. He is a make-up artist.” The first names presented were a selection of the most popular baby names from 1994 and 1984 which participants were likely to have been highly familiar with (sourced from Merry, 1995). Upon presentation of a picture and the accompanying sentences, participants were required to answer four questions relating to each characters’ probable salary (How much do you think [insert character name] earns each year?), leisure activities (What are [his/her] leisure activities?), job satisfaction (How satisfied do you think [he/she] is with [his/her] job?) and lifestyle (Briefly describe [his/her] personal life). Three different picture lists were created with the pictures presented in a different, but fixed, order in each list. Following this, three response booklets were prepared that matched the presentation order of the pictures. Note that the primary purpose of asking these questions was to focus participants’ attention on the pictures presented, and notably the job that each person was doing. However they were also a window to the views that participants hold about people in these different roles. Responses to these questions will be discussed after the results of Experiment 2.

#### Design and Procedure

In the judgment task, the two nouns were presented individually in the center of a computer screen. A role term was first displayed for 1000 ms, followed immediately by a kinship term (inter-stimulus interval of 0). This kinship term remained onscreen until a response was made. There followed a 500 ms delay before onset of the next trial. As described in Finnegan et al. (2015), the word pairs were divided into three fixed sets of blocks (with two of these chosen for each participant in the current study), with the order of the individual trials randomized separately for each participant. A button box was used to record participants’ responses, with one button clearly marked ‘Y’ for *yes* and another ‘N’ for *no*. Between the two blocks of trials, participants were asked to complete the picture task.

Participants were tested individually in a quiet laboratory. They were provided with written instructions that informed them to read each pair of words and decide (without excessive deliberation) whether the two terms could apply to the same person. Two examples of (definitional) word pairs were provided – one that required a *yes* response and one that required a *no* response. Participants were further informed that they would be required to make judgments about pictures between the first and second block of trials and told what this task entailed. The instructions and examples were then repeated verbally. Next, a short practice session using a representative sample of fillers and critical word pairs (not subsequently used in the experimental blocks) was given to familiarize the participants with the experimental task. Once familiarized with the procedure, participants were left alone to complete the judgment task.

### Results

#### Data Screening

In the analyses reported below, data for word pairs that contained the neutral term ‘adolescent’ were excluded because negative responses to such pairings (55% in Block 1, 33% overall) appeared to be based on considerations of age rather than gender. For instance, the pairing *adolescent/father* was much more difficult for participants to accept than *adolescent/brother*, despite both being possible combinations. In total, 1.32% of the data was removed for this reason.

#### Analysis

In both experiments accuracy of judgments and response times (RTs) were analyzed using two mixed-design analyses of variance (ANOVAs): firstly with participants treated as the random variable and secondly with items treated as the random variable. In the by-participants analysis (*F*_1_), the mixed ANOVA had three repeated factors – stereotype bias of the role name (Stereotype: Male/Female/Neutral), gender of the kinship term (Kinship term gender: Male/Female) and block of trials (Block: Block1/Block2). Participant Sex was included as a between-subjects factor. In the by-items analyses (*F*_2_), Stereotype was included as a between-items factor while Kinship term gender, Block and Participant Sex were included as within-item variables. Where sphericity was not satisfied, Greenhouse–Geisser (when 𝜀 < 0.75) or Huynh–Feldt (𝜀 > 0.75) corrected degrees of freedom and *p*-values are presented (as recommended by Girden, 1992). With all paired *t*-tests, within-subject or within-item effect sizes were estimated using Cohen’s *dz* while with independent-samples *t*-tests estimates of between-subject or between-item effect sizes were estimated using Cohen’s *d*.

#### Congruency

It is important to note that an interaction of Stereotype by Kinship term gender is an effect of Congruency, i.e., it is the combination of the levels of these two factors that give rise to the three critical conditions of congruent, incongruent and neutral. Therefore, Stereotype by Kinship term gender interactions are referred to as Congruency effects (though primarily in relation to the male and female stereotyped terms).

#### Accuracy

Analysis revealed a main effect of Stereotype, *F*_1_(1.67,46.67) = 6.27, *p* = 0.006, *F*_2_(2,32) = 9.59, *p* = 0.001, with higher accuracy to word pairs that contained a neutral role term (*M* = 94.3%), than those that contained male (*M* = 88.2%) or female-biased terms (*M* = 89.2%). A main effect of Block was also found, *F*_1_(1,28) = 6.90, *p* = 0.014; *F*_2_(1,32) = 17.73, *p* < 0.001, driven by a 3.5% increase in accuracy of critical pairings from Block 1 (88.8%), to Block 2 (92.3%). As anticipated, there was a main effect of Congruency, *F*_1_(1.18,33.08) = 14.76, *p* < 0.001; *F*_2_(2,32) = 67.55, *p* < 0.001, with significantly lower accuracy to stereotype incongruent word pairs (*M* = 79.80%), than to congruent (*M* = 97.15%) and neutral (*M* = 94.35%) pairings.

**FIGURE 1:**
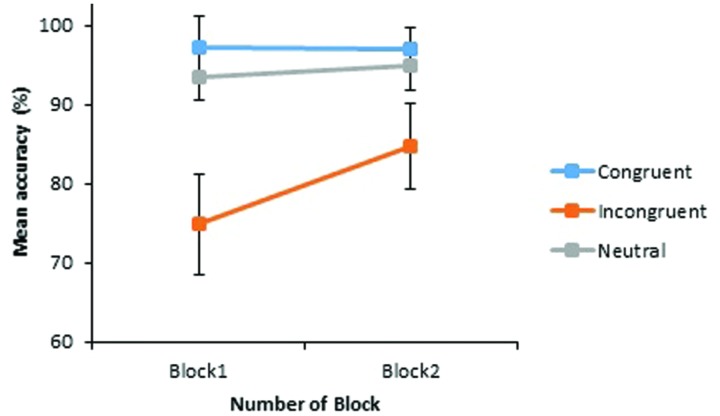
**Experiment 1: mean percentages of correct judgments to critical word pairs in Block 1 and Block 2.** The vertical axis begins at 60% while error bars indicate the 95% confidence intervals.

Importantly, an interaction of Congruency by Block was also found, *F*_1_(1.39,38.89) = 8.93, *p* = 0.002; *F*_2_(2,32) = 22.00, *p* < 0.001. This interaction was driven by a substantial 9.87% increase in accuracy for stereotype incongruent pairings across blocks, while accuracy to neutral and stereotype congruent pairings was high from the outset, with little room for improvement (see **Figure 1**).

The increase in accuracy to stereotype incongruent pairings across blocks was significant, *t*_1_(29) = 3.33, *p* = 0.002, *dz* = 0.61; *t*_2_(23) = 5.70, *p* < 0.001, *dz* = 1.16, revealing the efficacy of the counter-stereotypic picture task as a gender stereotype reduction strategy. However, despite this improvement across blocks, accuracy to stereotype incongruent word pairs remained significantly lower than accuracy to stereotype congruent, *t*_1_(29) = 3.10, *p* = 0.004, *dz* = 0.57; *t*_2_(23) = 9.56, *p* < 0.001, *dz* = 1.95, and neutrally rated word pairs, *t*_1_(29) = 2.60, *p* = 0.015, *dz* = 0.47; *t*_2_(44) = 6.65, *p* < 0.001, *d* = 2.00, by the end of the experiment. Thus, this picture training did not completely eradicate the effects of stereotype bias in this judgment task.

Next, an interaction of Participant Sex with Kinship term gender was revealed, *F*_1_(1,28) = 5.27, *p* = 0.029; *F*_2_(1,32) = 5.16, *p* = 0.030. Female participants displayed marginally higher accuracy in response to female kinship terms (88.5%) as opposed to male kinship terms (86.6%) while male participants displayed the opposite pattern, showing greater accuracy in response to male kinship terms (94.4%) than female kinship terms (92.8%). These mean values show that male participants were also more accurate than females on these kinship terms overall (93.6% vs. 87.6%).

A number of further effects involving Participant Sex emerged in the by-items analysis only^3^. A main effect of Participant Sex was first revealed, *F*_2_(1,32) = 104.01, *p* < 0.001, with male participants achieving much higher levels of accuracy than female participants overall (93.6% vs. 87.5%). There was also a highly significant interaction of Participant Sex by Congruency, *F*_2_(2,32) = 8.08, *p* = 0.001. While male participants outperformed females in each of the three congruency conditions, this difference was most apparent in response to stereotype incongruent pairings where male participants achieved an average accuracy score of 85.3% while female participants reached only 75.0%. Finally, there was a Participant Sex by Block interaction, *F*_2_(1,32) = 4.92, *p* = 0.034, with the accuracy of male participants increasing 2.4% across blocks, compared to 4.8% for female participants (although the females had more scope for improvement from Block 1). That said, the final accuracy of females was still lower than that of the males.

Reasons for this superior male performance remain unknown as (sex aside) there were no obvious differences between the male and female samples. The data suggests that male participants are more accepting of stereotype congruent pairings than past work suggests (Oakhill et al., 2005; Finnegan et al., 2015). This will be returned to in the General Discussion.

#### Response times

Response times below 150 ms, and above 4,000 ms were excluded from analysis (representing 0.92% of the total) along with times for all errors of judgment (representing a further 10.88%), totaling a loss of 11.8% of the data. These data points were replaced with the Participant by Block mean for each participant. Data points 2.5 standard deviation above or below the Participant by Block mean were replaced with the relevant upper or lower cut off point. Analyses were conducted as with the accuracy data.

A main effect of Stereotype was found in the by-participants analysis, along with a marginally significant effect in the by-items analysis, *F*_1_(2,56) = 5.50, *p* = 0.007; *F*_2_(2,32) = 3.00, *p* = 0.064, with faster RTs to word pairs that contained a neutral role term (*M* = 828 ms), than those that contained male-biased (*M* = 850 ms) or female-biased terms (*M* = 889 ms). A main effect of Block was also revealed, *F*_1_ (1,28) = 15.50, *p* < 0.001; *F*_2_(1,32) = 97.60, *p* < 0.001, with RTs decreasing 143 ms from Block 1 to Block 2 (927 ms vs. 784 ms respectively). Again, there was a main effect of Congruency, *F*_1_(1.33,37.12) = 12.31, *p* < 0.001; *F*_2_(2,32) = 11.62, *p* < 0.001, with fastest RTs observed in response to stereotype congruent word pairs (*M* = 815 ms), followed by neutral (*M* = 829 ms) and incongruent pairings respectively (*M* = 920 ms).

**FIGURE 2:**
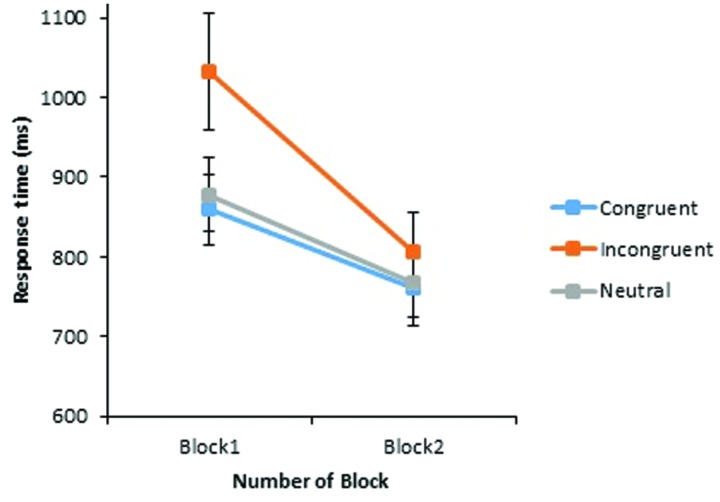
**Experiment 1: mean response times (in milliseconds) of correct judgments to critical word pairs across Block 1 and Block 2.** The vertical axis begins at 600 ms while error bars indicate the 95% confidence interval.

Importantly, a significant interaction between Block and Congruency also emerged, *F*_1_(2,56) = 4.87, *p* = 0.011; *F*_2_(2,32) = 5.27, *p* = 0.010. As can be seen in **Figure 2**, RTs decreased across all conditions from Block 1 to Block 2, with the greatest reduction found in response to stereotype incongruent pairings (225 ms). This was found to be a significant improvement across blocks, *t*_1_(29) = 4.23, *p* < 0.001, *dz* = 0.77; *t*_2_(23) = 7.89, *p* < 0.001, *dz* = 1.61. Furthermore, by the end of the experiment, there was no significant difference between RTs to stereotype incongruent and stereotype congruent, *t*_1_(29) = 1.59, *p* = 0.122; *t*_2_(23) = 1.65, *p* = 0.112, or neutral pairings, *t*_1_(29) = 1.36, *p* = 0.183; *t*_2_(44) = 1.41, *p* = 0.167. Overall, the RT data provide further strong support for the use of counter-stereotypical pictures as an effective stereotype-reduction strategy. However, it should be noted that past results suggest that this improvement across blocks is also likely due in part to practice effects (Finnegan et al., 2015).

A main effect of Participant Sex was also observed, *F*_1_(1,28) = 5.64, *p* = 0.025; *F*_2_(1,32) = 93.24, *p* < 0.001, with male participants typically much slower to respond than female participants (925 ms vs. 786 ms). An interaction of Participant Sex with Kinship term gender also emerged, *F*_1_(1,28) = 14.23, *p* = 0.001; *F*_2_(1,32) = 9.09, *p* = 0.005. Again, female participants responded faster to female kinship terms over male kinship terms (765 ms vs. 807 ms respectively), while male participants responded faster to male kinship terms over female kinship terms (892 ms vs. 958 ms respectively). These means also reveal that female participants responded faster than male participants in both cases (786 ms vs. 925 ms), a finding which may shed some light on the lower accuracy scores achieved by females in this study; it is possible that accuracy performance may have deteriorated for the sake of faster responding.

#### Fillers – Accuracy

As responding to the filler trials was not the main focus of this research, tests of significance were not carried out on these data; only a descriptive analysis is presented (with one exception below). Performance on the filler trials was somewhat variable, with an average of 93.0% accuracy on definitionally matching word pairs versus an average of 87.28% on definitionally mismatching word pairs across the experiment. As in Finnegan et al. (2015), accuracy of responses to definitionally mismatching word pairs was lower to trials involving male terms (*M* = 80.61%) than female terms (*M* = 93.94%). This pattern was previously thought to result from the generic interpretation of certain terms such as *host* (0.45% accuracy across blocks) or *steward* (0.25% accuracy across blocks) which have female-specific counterparts (i.e., *stewardess* and *hostess*) and which should, therefore, be taken as male specific. However, to investigate whether this effect was driven by male participants (for whom these pairings are more self-relevant than females) we conducted a mixed ANOVA on the definitionally mismatching responses. A significant interaction of Participant sex by Definitional gender was indeed found, *F*_1_(1,28) = 4.71, *p* = 0.038; *F*_2_(1,58) = 5.61, *p* = 0.021 with males achieving lower accuracy to definitionally male role nouns when presented with a female kinship term (76.0%) than females did to these pairings (84.7%), while both sexes scored more similarly on the definitionally female terms paired with male kinship terms (92.9% vs. 94.9% respectively). While this data suggests that poor performance on the definitionally mismatching pairings was due to the male participants struggling with the male mismatch terms, this interaction was not replicated in the reaction time data described below (*ps* > 0.3). Nor was it replicated in the accuracy or reaction time data of Experiment 2 (*p*s > 0.5). For this reason we maintain that it is likely the generic interpretation of certain definitionally male terms that is driving poor performance to definitionally mismatching pairings.

#### Fillers – Response Times

Average RTs to definitionally matching word pairs were found to be faster than those to definitionally mismatching word pairs (888 ms vs. 950 ms respectively). RTs were also faster in response to female pairings over male in both the definitionally matching (862 ms vs. 914 ms respectively) and mismatching cases (910 ms vs. 989 ms respectively). These findings reflect the accuracy data, with longer processing of male mismatching pairs likely to reflect participants’ deliberation over terms that are masculine by definition but often used generically in reference to both sexes.

### Discussion

Overall, Experiment 1 provides preliminary evidence for the use of counter-stereotypical pictures as an effective strategy for reducing the immediate activation of gender stereotypes when gender-biased role terms are read. Both accuracy and reaction times to stereotype incongruent word pairs significantly improved from Block 1 to Block 2 following the counter-stereotypic picture task. While accuracy remained significantly lower to the incongruent pairs than to the stereotype congruent and neutral pairings in Block 2, RTs in Block 2 were similar in all three conditions.

It is hypothesized that exposure to the counter-stereotypical pictures triggered participants’ world knowledge that, although there is a strong gender bias associated with certain social roles in society, nowadays both men and women can and do fulfill these roles. The activation of this knowledge is then thought to have helped participants overcome stereotype application in the second block of judgment trials.

Before accepting this picture training as a successful means of stereotype reduction, a control condition against which these results could be compared was required so as to verify that the counter-stereotype manipulation of Experiment 1 was indeed the reason for the improved task performance in Block 2, rather than simply looking at pictures of people carrying out jobs and answering questions about these people. In Experiment 2, therefore gender *stereotypical* pictures replaced the counter-stereotypical pictures in the picture task. If there are stereotype-related effects from processing the pictures, Experiment 2 should see the maintenance of (as opposed to the weakening of) the gender biases associated with many occupational terms in English.

## Experiment 2

By providing participants with pictures of people working in gender stereotypical roles, Experiment 2 sought to reinforce participants’ world knowledge that women are typically associated with a certain set of roles (e.g., *beautician, secretary*), and men are typically associated with another set (*pilot, mechanic*). The experimental design was exactly as outlined in Experiment 1, but with the counter-stereotypical pictures replaced by stereotypical pictures. The rationale for Experiment 2 was that attending to these gender-stereotypical pictures would lead to deeper adherence to gender biases in the judgment task. Therefore, if there was no improvement in response to stereotype incongruent trials from Block 1 to Block 2, it could be confidently assumed that the reduction in stereotype bias across blocks in Experiment 1 was associated with the presentation of counter-stereotypical pictures.

As in Experiment 1, it was hypothesized that participants would initially respond more slowly and less accurately to trials with stereotype incongruent word pairs (e.g., *nurse/father*) than to stereotype congruent word pairs (*nurse/mother*) in Block 1. However, unlike Experiment 1, it was hypothesized that the processing cost associated with the stereotype incongruent condition in Block 1 would not be attenuated in Block 2 following presentation of the stereotype congruent pictures.

### Method

#### Participants

The participants were 34 monolingual native English speaking students (19 female, 15 male) from the University of Sussex. Participants’ ages ranged from 18 to 32 years (*M*: 21.23; SD: 4.53). They received either £6 or 4 course credits for taking part in the session which lasted approximately 45 min.

#### Materials

The same materials and instructions were employed as in Experiment 1, aside from a different set of pictures (and accompanying booklets) for the picture task. The pictures all depicted men and women working in a stereotypical job environment and were accompanied by two sentences introducing the character and stating their job, e.g., *This is Rebecca. She is a make-up artist* or *This is Christopher. He is a bricklayer*. As a reminder, the stereotypic pictures used in this study were previously rated for similarity (to the counter-stereotyped pictures) and realism in the pilot study.

#### Design and Procedure

The design and procedure were identical to those for Experiment 1, but with participants answering questions about pictures of people working in stereotypical roles as opposed to counter-stereotypical roles.

### Results

#### Data Screening

In this Experiment, the neutral term ‘adolescent’ was replaced with the term ‘swimmer’ therefore data for all neutral items were included in the analysis. Accuracy of and RTs for judgments were analyzed as in Experiment 1.

#### Accuracy

**FIGURE 3:**
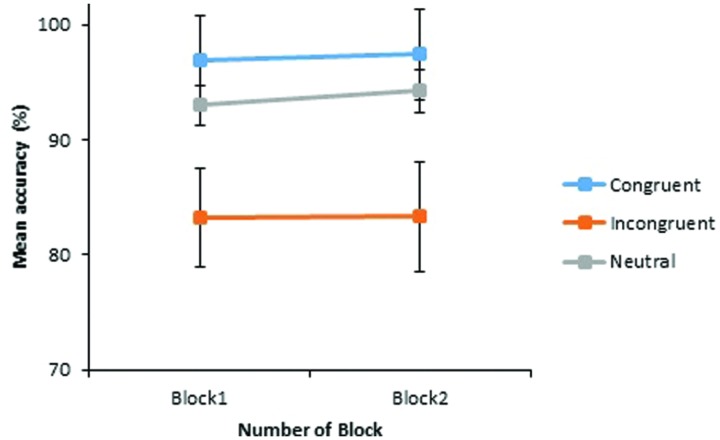
**Experiment 2: mean percentages of correct judgments to critical word pairs in Block 1 and Block 2.** The vertical axis begins at 70% while error bars indicate the 95% confidence interval.

A main effect of Stereotype was found, which was significant by participants and marginally significant by-items, *F*_1_(1.30,41.61) = 7.81, *p* = 0.004; *F*_2_(2,33) = 3.10, *p* = 0.059, with greater accuracy for neutral role nouns (*M* = 93.1%), than male-biased (*M* = 90.7%) or female-biased terms (*M* = 88.8%). A main effect of Congruency was also revealed, *F*_1_(1.03,33.07) = 12.47, *p* = 0.001; *F*_2_(2,33) = 55.04, *p* < 0.001, with significantly higher accuracy to stereotype congruent (*M* = 97.0%) and neutral (*M* = 93.1%) word pairs, than to stereotype incongruent pairings (*M* = 83.3%). However, no significant effect of Block was found, *F*_1_(1,32) = 0.89, *p* = 0.351; *F*_2_(1,33) = 0.67, *p* = 0.417, with accuracy increasing just 0.5% across the two blocks (Block 1 *M* = 90.6% vs. Block 2 *M* = 91.1%). Importantly, there was also no significant interaction of Congruency by Block, *F*_1_(2,64) = 1.05, *p* = 0.357; *F*_2_(2,33) = 0.74, *p* = 0.490, with responding across conditions shown in **Figure 3**.

Accuracy for stereotype incongruent pairings failed to significantly increase across the blocks [(+0.26%), *t*_1_(33) = 0.10, *p* = 0.918; *t*_2_(23) = 0.15, *p* = 0.880], suggesting that the stereotypical picture training did indeed maintain stereotype biases. However, it is worth nothing that Block 1 accuracy to incongruent pairings in this study was considerably higher than Block 1 accuracy to incongruent pairings in Experiment 1 (83.21% vs. 74.86% respectively), thus leaving less scope for improvement in the current study. This issue is returned to in the General Discussion. Accuracy also remained significantly poorer for stereotype incongruent pairings than for neutral [*t*_1_(33) = 3.718, *p* = 0.001, *dz* = 0.64; *t*_2_(23) = 6.70, *p* < 0.001, *dz* = 1.37] and stereotype congruent pairings [*t*_1_(33) = 3.32, *p* = 0.002, *dz* = 0.57; *t*_2_(23) = 8.64, *p* < 0.001, *dz* = 1.76] by the end of the experiment.

Finally, two effects involving Participant Sex were found in the by-items analysis only. There was a main effect of Participant Sex, *F*_2_(1,33) = 165.93, *p* < 0.001, with female participants achieving much higher levels of accuracy than male participants overall (94.9% vs. 86.5%). There was also a Participant Sex by Congruency interaction, *F*_2_(2,33) = 22.14, *p* < 0.001, with female participants outperforming males in each of the congruency conditions, but particularly in response to incongruent pairings (89.15% vs. 75.85% respectively). In contrast to Experiment 1, it was now females who outperformed males in accuracy performance. The reason(s) for this contrasting performance between both sexes remain(s) unclear as again there were no obvious differences between the two samples.

#### Response Times

**FIGURE 4:**
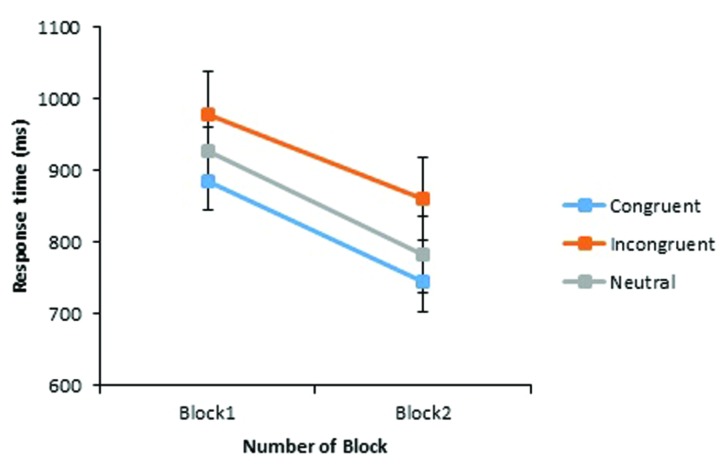
**Experiment 2: mean response times (in milliseconds) of correct judgments to critical word pairs across Block 1 and Block 2.** The vertical axis begins at 600 ms while error bars indicate the 95% confidence interval.

Response times below 150 ms, and above 4,000 ms were excluded from analysis (representing 1.77% of the total data) along with times for all errors of judgment (representing a further 12.85%), totaling a loss of 14.61% of the data. These data points were replaced as in Experiment 1. A significant main effect of Congruency was found, *F*_1_(2,64) = 15.18, *p* < 0.001; *F*_2_(2,33) = 7.22, *p* = 0.003, with fastest RTs to stereotype congruent word pairs (*M* = 817 ms), followed by neutral (*M* = 862 ms) and incongruent pairings respectively (*M* = 920 ms). A main effect of Block was also observed, *F*_1_(1,32) = 14.56, *p* = 0.001; *F*_2_(1,33) = 130.93, *p* < 0.001, with average RTs decreasing 128 ms from Block 1 to Block 2. As with the accuracy data, there was no evidence of a Congruency by Block interaction, *F*_1_(1.82,58.12) = 0.38, *p =* 0.663; *F*_2_(2,33) = 0.01, *p* = 0.988, as RTs were found to decrease to a similar extent across blocks in all three congruency conditions (see **Figure 4**). While these improvements were each statistically significant (*p* < 0.03), they are taken as evidence for task habituation, as participants got progressively faster at responding to all critical word pairs as the task progressed, without any equivalent increase in accuracy performance across critical trials. A significant difference between RTs to stereotype incongruent and congruent pairs remained at the end of the experiment, *t*_1_(33) = 2.90, *p* = 0.007, *dz* = 0.50; *t*_2_(23) = 2.82, *p* = 0.010, *dz* = 0.57, and also between stereotype incongruent and neutral pairings, *t*_1_(33) = 2.15, *p* = 0.039, *dz* = 0.37; *t*_2_(23) = 2.13, *p = 0*.044, *dz* = 0.43.

An interaction of Participant Sex by Kinship term gender also emerged, *F*_1_(1,32) = 8.17, *p* = 0.007; *F*_2_(1,33) = 8.32, *p* = 0.007, with female participants faster when responding to female kinship terms over male kinship terms (796 ms vs. 855 ms respectively). Male participants were faster at responding to male kinship terms than female kinship terms (885 ms vs. 936 ms respectively), although they were slower than females at both.

There was also a main effect of Participant Sex in the by-items analysis only, *F*_1_(1,32) = 0.71, *p* = 0.406; *F*_2_(1,33) = 19.34, *p* < 0.001, with male participants slower at responding than female participants overall (880 ms vs. 815 ms respectively). Finally, a significant three-way interaction of Block by Congruency by Participant Sex was found in the by-participants analysis, *F*_1_(1.82,58.12) = 4.21, *p* = 0.023; *F*_2_(2,33) = 1.62, *p* = 0.214. This interaction was driven by performance to stereotype incongruent pairings. Females were faster to respond to these pairings than males in Block 1 by just 7 ms but this improvement jumped to 227 ms in Block 2 as female participants outperformed the males.

#### Fillers – Accuracy

Performance on the definitionally matching word pairs revealed a high mean accuracy score of 93.6% across blocks, with similar performance on both the male (94.2%) and female pairings (92.9%). However, performance was poorer on the definitionally mismatching word pairs (*M* = 83.7%). Average accuracy to definitionally female pairings was high at 91.3%, but dropped to 76.2% for the definitionally male pairings. Again, it is hypothesized that this difference in accuracy performance is due to the generic interpretation of certain male terms that are in fact male-specific by definition.

#### Fillers – Response Times

The RT data tell a similar story to the accuracy data. Reaction times to both male and female definitionally matching word pairs were similar (926 ms for the male versus 880 ms for the female pairs) with an average RT of 903 ms across blocks. Average RTs in the definitionally mismatching condition were slower, at 982 ms. Female mismatching pairings were responded to faster (943 ms) than male mismatching pairings (1022 ms) in general, again likely because participants considered that certain male terms can be used generically despite their gender specific definitions.

### Discussion

This control experiment sought to maintain the stereotypical gender bias associated with certain role terms in English by presenting participants with pictures of men and women working in gender stereotypical roles. The hypothesis that accuracy of judgments on the stereotype incongruent word pairs would not improve across blocks in the judgment task was indeed supported. However, RTs to stereotype incongruent pairings *were* found to speed up across blocks in all congruency conditions. This pattern of results suggests that participants were benefitting from a practice effect and naturally speeding up at the task as it progressed. While RTs in Experiment 2 improved consistently across all conditions, RTs to the stereotype incongruent pairings in Experiment 1 decreased more sharply, with final RTs in line with those of stereotype congruent and neutral pairings.

As accuracy of stereotype incongruent trials did not significantly increase across blocks in Experiment 2, it is concluded that processing and making judgments about stereotype-consistent pictures did not help participants to overcome gender stereotype biases. It, therefore, appears that processing of specifically counter-stereotypical information in Experiment 1 was the reason for the improved performance on counter-stereotypical pairs in Block 2 of the judgment trials. We conclude that increasing exposure to counter-stereotype pictures is a useful means of moderating the effects of immediate gender activation in the judgment task.

### Questionnaire Analysis

Participants were asked four questions in relation to each of the pictures presented to them in Experiments 1 and 2. Two of the questions required responses to be made along a Likert scale (earnings and job satisfaction) while two were open-ended (lifestyle and personal life). Responses to the latter two questions varied greatly in the level of detail and content provided by participants and were subsequently rated by two independent raters (one male, one female) along various dimensions described further below. The raters first analyzed the data independently and then met to compare results and try to reach a consensus on conflicting ratings. All inconsistencies were resolved after discussion so all data was kept. This rating procedure allowed for a statistical analysis of the subjective responses provided by participants in questions 3 and 4.

All questionnaire data was analyzed using a 2 (Participant sex: male, female) × 2 (Experiment: stereotype, counter stereotype) × 2 (Character: male, female) independent ANOVA.

**Question 1**. Earnings: How much do you think [insert character name] earns each year? Response options were on a 6-point scale, ranging from (1) < £10,000 to (6) > £50,000.

There was a significant main effect of Experiment, with those depicted in counter-stereotypical roles (*M* = 3.42) thought to earn more than those in stereotypical roles (*M* = 3.12), *F*(1,120) = 6.58, *p* = 0.012. There was also a significant main effect of Character with male characters deemed to earn more (*M* = 3.40) than female characters (*M* = 3.21), *F*(1,120) = 4.52, *p* = 0.036.

A significant interaction of Experiment by Character [*F*(1,120) = 95.58, *p* < 0.001] also emerged, with males working in stereotypical roles thought to earn more than males working in counter-stereotypical roles (*M* = 3.73 vs. *M* = 3.08 respectively) while females working in stereotypical roles were thought to earn less than females working in counter-stereotypical roles (*M* = 2.65 vs. *M* = 3.77 respectively). This pattern of results may reflect the high status associated with some of the typically male jobs used in this study (e.g., surgeon, judge, architect) compared to the lower status associated with many of the typically female jobs used (e.g., cleaner, hairdresser, au-pair).

**Question 2**. Job satisfaction: How satisfied do you think [he/she] is with [his/her] job? Response options to the above question were on a 5-point scale ranging from (1) ‘Extremely Satisfied’ to (5) ‘Extremely Dissatisfied.’

No significant differences emerged in relation to job satisfaction, with ratings falling between 2.09 and 2.14 across all comparisons.

**Question 3**. Leisure: What are [his/her] leisure activities?

As mentioned earlier, responses to questions 3 and 4 were rated along various dimensions by two raters. The dimensions for question 3 were (1) Male typical vs. Female typical (2) Physically oriented or Mentally oriented (3) Social or Solitary activities and (4) High vs. Low cost.

Male typical vs. Female typical. Responses were rated according to the scale: 1 = Male-typical, 2 = Neutral, 3 = Female-typical.

There was a main effect of Character with female characters deemed to engage in more female-typical leisure activities and male characters thought to engage in more male-typical leisure activities (*M* = 2.17 vs. *M* = 1.69 respectively), *F*(1,120) = 143.22, *p* < 0.001. There was also a significant interaction of Experiment by Character [*F*(1,120) = 146.025, *p* < 0.001], with males in stereotypical roles judged as engaging in male-typical activities (*M* = 1.43) while females in stereotypical roles were judged as engaging in female-typical activities (*M* = 2.40). However, males and females in counter stereotypical roles were judged as having similarly rated leisure activities, close to the neutral point of 2 (*M* = 1.95 vs. *M* = 1.94 respectively).

Physically oriented vs. Mentally oriented. Responses were rated according to the scale: 1 = Physical, 2 = Physical and Mental, 3 = Mental.

There was a main effect of Character with female characters judged to engage in more mentally oriented leisure activities than male characters (*M* = 2.28 vs. *M* = 2.14), *F*(1,120) = 8.50, *p* = 0.004. There was also a significant interaction of Experiment by Character [*F*(1,120) = 26.72, *p* < 0.001], with females in stereotypical roles judged as engaging in more mentally oriented leisure activities than males (*M* = 2.40 vs. *M* = 2.03). However, males in counter stereotypical roles were judged as engaging in more mentally-oriented leisure activities than females in these same roles (*M* = 2.26 vs. *M* = 2.15).

Social vs. Solitary. Responses were rated according to the scale: 1 = Social, 2 = Neutral, 3 = Solitary. No significant differences were found to emerge in this category, with typical ratings falling close to 2 (i.e. neutral) across all comparisons. High cost vs. Low cost. Responses were rated according to the scale: 1 = Expensive, 2 = Reasonable, 3 = Cheap.

A main effect of Character was found with female characters judged to engage in somewhat cheaper leisure activities than male characters (*M* = 2.33 vs. *M* = 2.23 respectively), *F*(1,120) = 4.24, *p* = 0.042. There was also a marginal interaction of Participant sex by Character [*F*(1,120) = 3.85, *p* = 0.052] with male participants judging female characters as engaging in cheaper activities than male characters (*M* = 2.41 vs. *M* = 2.21), while female participants gave more similar ratings across female and male characters (*M* = 2.26 vs. *M* = 2.25 respectively).

**Question 4**. Briefly describe [his/her] personal life.

Responses to Question 4 were first rated along the dimensions (1) Traditional vs. non-traditional and (2) Happy vs. unhappy.

Traditional vs. Non-traditional. Responses were rated according to the scale: 1 = Traditional, 2 = Neutral, 3 = Non-traditional.

There was a main effect of Experiment with those working in stereotypical roles judged to lead more traditional personal lives than those working in counter-stereotypical roles (*M* = 1.59 vs. *M* = 1.72 respectively), *F*(1,120) = 9.51, *p* = 0.003. There was also a significant interaction of Experiment by Participant Sex [*F*(1,120) = 5.48, *p* = 0.021], as female participants deemed that those working in stereotypical roles lived more traditional roles than those working in counter-stereotypical roles (*M* = 1.56 vs. *M* = 1.78 respectively) while male participants judged that those in stereotypical and counter-stereotypical roles lead similarly traditional lives (*M* = 1.63 vs. *M* = 1.66 respectively).

A significant interaction of Experiment by Character was also found [*F*(1,120) = 21.37, *p* < 0.001], with females in counter stereotypical roles thought as leading a more traditional personal life than males working in counter stereotypical roles (*M* = 1.63 vs. *M* = 1.81 respectively). However, females in stereotypical roles were judged as leading a more non-traditional personal life than males working in stereotypical roles (*M* = 1.70 vs. *M* = 1.47 respectively). That said, it is worth noting that all mean values fell between the rating points of Traditional and Neutral as opposed to Non-traditional.

No significant differences were found to emerge with ratings falling between 1.80 and 1.90 across all comparisons, i.e., between the points of Happy (1) and Neutral (2). Happy vs. Unhappy. Responses were rated according to the scale: 1 = Happy, 2 = Neutral, 3 = Unhappy.

Overall, the picture booklets provide interesting supplementary data on the perception of men and women working in stereotypical and counter-stereotypical occupational roles. While integrating the findings into current social psychological literature is beyond the scope of this article, future research could further examine the themes which have emerged in our analysis.

## General Discussion

In an effort to build on past research aimed at identifying strategies for overcoming stereotypes and prejudice, the current studies investigated the use of counter-stereotype information as a moderator of gender stereotype use. Experiment 1 involved presenting participants with pictures of people working in gender counter-stereotypical roles, and answering questions about the characters in these pictures. It was hypothesized that the questions would focus participants’ attention on the characters presented (specifically their jobs), and that the pictures would be a salient reminder that people can work in gender atypical roles. It was found that accuracy of response to stereotype incongruent pairings did significantly increase after this picture training but importantly did not improve in Experiment 2; a control experiment in which participants were presented with pictures of people working in gender stereotypical roles. RTs decreased across blocks in both Experiments 1 and 2, independently of the type of picture training received. We posit that the decrease in RTs in Experiment 1 was due to the counter-stereotype picture manipulation, however, in Experiment 2, the lack of improvement in the accuracy data suggest this latter decrease was due to practice effects. Indeed this Experiment 2 data reflects unpublished findings from our lab in which RTs improved across blocks in the absence of any experimental manipulation whereas accuracy scores did not change.

However, interpretation of the results is not wholly straightforward as Block 1 accuracy was higher in Experiment 2 (83.21%) than Experiment 1 (74.86%). The reason(s) for such Block 1 differences remain unknown as both experiments were identical up until the picture task (between Block 1 and Block 2 of the judgment trials), and there were no discernible differences between the participant samples. This situation resulted in less scope for improvement across Blocks in Experiment 2 and final accuracy levels were similar across experiments. It is, therefore, not completely clear how the different picture strategies would have affected Block 2 performance if initial performance had been more similar. This same issue arose in Finnegan et al. (2015) and suggests that the judgment paradigm may benefit from further scrutiny when used in between-subject designs. That said, we argue that this Block 1 variability is not crucial to the conclusions we have drawn, as we were primarily interested in participants’ response to the counter-stereotype information: specifically, whether this information would lead to a revision of participants stereotyped associations, or whether it would be ignored. While the results should be interpreted with caution because of the differential Block 1 performance, it appears that activating counter-stereotype gender associations did lead to a revision of participants’ stereotyped beliefs and ultimately helped them to control stereotype use in the judgment task.

Effects of Participant Sex were not anticipated in this research based on previous findings (Oakhill et al., 2005; Finnegan et al., 2015). However, in Experiment 1 males showed higher accuracy scores to critical trials, yet female participants were faster to respond. As such it cannot be ascertained whether female participants forsake accuracy so as to complete the test quickly (regardless of inaccurate responding) or whether they were simply weaker at recognizing and overcoming stereotype biases than the male participants. In Experiment 2 female participants were found to have higher levels of accuracy to critical trials than males and were also faster at responding. Therefore, in contrast to Experiment 1, the latter results suggest females are better at quickly recognizing and overcoming occupational stereotypes than males. Reasons for the differential performance of males and females across experiments remain unknown. While it is possible that the counter stereotype training task may have induced male participants to think more about their responding than in the stereotypical condition, it is unclear why female participants would not also respond to this training task.

The use of overt and striking counter-stereotype stimuli as part of the training in Experiment 1 provides evidence for the conversion theory of stereotype change, i.e., that the stereotypes can change rapidly on encountering a few, striking, counter-stereotype exemplars. It is also possible that bookkeeping processes may have played a role. As 24 pictures of people working in counter-stereotypical roles were presented, stereotype change is likely to have been somewhat incremental and become stronger as the participants proceeded through the pictures and questions. However, these findings provide less support for the subtyping theory of stereotype change which stipulates that the original stereotype can be protected through the formation of new categories to account for counter-stereotypical information. Although it cannot be definitively ruled out that participants used subtyping processes to account for the counter-stereotype exemplars, it seems unlikely that such a number of counter-stereotype exemplars could be easily rationalized in this way. On the contrary, the findings suggest that stereotypes can be weakened with sufficient category variation (Operario and Fiske, 2004).

Participants who received the counter-stereotype pictures seem to have been reminded that stereotypes are maladaptive forms of categories in that their content is not always accurate. Indeed, explicit training strategies such as this, in which counter-stereotype saliency is increased, may simply remind participants of specific things they already know, e.g., that a woman can be a surgeon and a man can be a nurse. It is logical to assume that with more frequent exposure, counter-stereotypic associations should become more accessible and the issue of gender ‘atypical’ roles may become obsolete. If true, this approach shows promise for inducing long-term stereotype change and could, with time, result in perceivers delaying the assignment of gender to a referent when gender-biased occupational terms are encountered (and hold back until more definitive gender information is supplied).

Repeated exposure to cultural images that reinforce automatic stereotypical or prejudiced associations means that these biases can become entrenched and difficult to overcome. Although people can often control and prevent the influences of stereotypes on overt behavior, such correctional efforts can be cognitively demanding and rely on factors such as a perceivers’ awareness, motivation and cognitive resources, each of which can be easily undermined. Ideally, stereotype reduction research would aim to combat the initial activation of stereotypes as opposed to controlling the subsequent influence of these biases on behavior (Bodenhausen and Macrae, 1998; Gawronski et al., 2008). Also, although counter-stereotypes are by definition not highly accessible, and are unlikely to be implicitly activated and influence behavior to the extent that stereotypic associations do (Blair et al., 2001), their accessibility and influence can be increased given certain conditions. The use of counter-stereotypical pictures as a stereotype reduction manipulation is an example of a strategy that could be easily applied at a broad, societal level so as to increase exposure to counter-stereotype exemplars, and consequently instigate real change in the cognitive representations of gender-biased terms. For instance, it seems likely that frequent depiction of men and women working in gender atypical roles in educational material would effect change in students’ cognitive representations of gender to accommodate this information^4^. Gender-fair pictures could also be used in other contexts where occupational stereotypes may be in use, e.g., in certain job advertisements. Future research could aim to evaluate the efficacy of exposure to counter-stereotypical pictures across a variety of different contexts in both the short- and longer-term.

Macrae and Bodenhausen (2000) suggest that there is an over-reliance on verbal category labels in research investigating the process of category activation. They caution that this over-reliance is problematic as in reality people are complex stimuli that can be classified by perceivers along multiple dimensions. Consequently, it cannot be assumed that the processing of verbal labels equates to the processes involved in person perception. The counter-stereotype picture training of Experiment 1 support this call of Macrae and Bodenhausen (2000) to move beyond the use of verbal stimuli (category labels) and to use more realistic stimuli. As stereotype reduction interventions are often detached from a ‘real-life’ context, doubt is cast on their usefulness beyond a laboratory setting (Lenton et al., 2009; Paluck and Green, 2009). While future research would undoubtedly benefit from an investigation of the cognitive processes involved in stereotype activation and application upon encountering real people, the use of pictures of people at work is a step in the right direction toward identifying further effective means of stereotype reduction with a training higher in ecological validity than many others.

Although the results of this research provide strong support for the malleability of gender stereotype biases, they also echo previous studies using this judgment paradigm that document the persistency of stereotyping effects. We found that the processing of stereotype incongruent pairings rarely achieved the same level of effortlessly fast and accurate responding as that of stereotype congruent and neutral pairings. This same level of success (or lack of complete success) at overcoming occupational gender biases was previously found with strategies that included explicitly reminding participants that many jobs are not gender differentiated these days (Oakhill et al., 2005), and providing social consensus feedback that suggested past participants had no problem accepting stereotype incongruent pairings as correct (i.e., they were gender fair in their responding; Finnegan et al., 2015). Thus it appears that gender biases associated with social and occupational role nouns are deeply ingrained and difficult to overcome. Also, the fact that stereotypes are activated even when detrimental to task performance is further evidence that these biases are likely to be automatically activated.

In agreement with the assertion of past authors (e.g., Cohen, 1994; Lai et al., 2014) that it is important to include estimates of effect size in stereotype reduction research so as to assess whether an intervention has practical significance, we compared effect sizes of the current study with the above-mentioned research by Finnegan et al. (2015). We found that the current research led to larger effect sizes for the increase in accuracy to incongruent pairings in the by-participants (*dz* = 0.61 vs. *dz* = 0.35) and by-items (*dz* = 1.16 vs. *dz* = 0.87) analyses respectively. Effect sizes were more similar in the RT data, with larger effects found in the current work in the by-participants data (*dz* = 0.77 vs. *dz* = 0.61) while the opposite pattern was found in the by-items analyses (*dz* = 1.61 vs. *dz =* 1.88). However, as the work of Finnegan et al. (2015) involved three blocks of trials as opposed to two, it is likely to have benefitted more from practice effects. This comparison thus suggests that exposure to pictures of people working in counter-stereotypical occupations can lead to more reduced levels of stereotype application than feedback based on social norm information, at least in the short-term, and on the judgment task of Oakhill et al. (2005). The value of including effect sizes in research on stereotype and prejudice reduction can be seen in such comparisons and we recommend that it become standard procedure in this domain^5^.

Overall, the case for reducing gender biases in relation to occupational stereotypes is not a trivial one. On the contrary, such efforts may have important implications for career choice, as exposure to gender stereotypes can influence preference toward jobs and activities from an early age. For instance, Liben et al. (2002) found that children aged 6–11 have quite fixed opinions about whether certain roles can be applied to women and men, typically stating that doctors are men and nurses are women. Moreover, Gottfredson’s (1981, 2005) theory on career development asserts that children around 6 years-old begin to lose interest in occupations that are not in line with their gender self-concept. Such research suggests that gender stereotypes lead to inequality by artificially limiting the choices on offer to both sexes. As such, it is imperative to devise interventions that challenge people’s gendered perceptions and ultimately lead to a reduction in gender stereotyping. Increased exposure to counter-stereotypical exemplars could be a practical step toward achieving this aim.

## Conflict of Interest Statement

The authors declare that the research was conducted in the absence of any commercial or financial relationships that could be construed as a potential conflict of interest.

## Footnotes

1 Lai et al. (2014) point out that this comparative study allowed for inferences about the interventions to be made in a highly specific experimental context only. For instance, in all cases the Implicit Association Test (IAT) or Multi-Category IAT was used as the main dependent variable, while a number of specific criteria had to be fulfilled in order to win the research contest (as opposed to inclusion in the study) e.g., at least 85% of participants had to finish the intervention in 5 min or less. Such criteria may have made it more likely to find an effect using certain interventions over others. For instance, evaluative conditioning may be more effective over longer intervention formats (Bar-Anan et al., 2010).

2 It was hypothesized that the role terms that appeared in the picture booklet would elicit a higher level of accuracy for the stereotype incongruent pairs (i.e., judgments that the two words can refer to the same person) and lower response times in Block 2 of the judgment task than those that did not appear in the picture booklet (as the pictures explicitly depicted a person of counter-stereotypical gender fulfilling the role). However, it was found that accuracy to both sets of terms was identical (at 88%) while RTs were somewhat slower for the role nouns that had previously appeared in the booklet (*M* = 731 ms) compared to those that did not (*M* = 686 ms), although this difference was not significant, *t*(22) = 1.34, *p* = 0.193. Although this latter trend was not anticipated, we suggest that the pictures participants saw may have induced them to think more about these specific occupations as they arose in the judgment task thus leading to increased reaction times.

3 Given that there were many fewer participants than items in this experiment (30 participants vs. 304 item pairs per participant) it is highly likely that this effect was only significant by-items because the standard errors of the condition means are likely to be much lower in the by-items analysis than in the by-participants analysis, if the variances are roughly equal. For instance, the average standard deviation of responses to critical word pairs was 16.3% in the by-participants data while just 5.3% in the by-items analysis. As a similar imbalance between participant numbers and item numbers runs throughout both studies in this article this pattern (a significant effect by-items but not by-participants) frequently recurs.

4 Although some studies report that school books have become more gender fair across recent years (e.g., Diekman and Murnen, 2004; Moser and Hannover, 2013), effects of these changes on longer term cognitive representations of gender remain unknown.

5 However, note that effect sizes like Cohen’s *dz* which control for individual differences can be inflated compared to the effect sizes reported in between-subject designs where individual differences cannot be controlled (Dunlap et al., 1996; Lakens, 2013). Therefore caution should be exercised in comparing these effect sizes with training strategies used in conjunction with between-subject studies.
